# Association of Gestational Age at Birth With Subsequent Neurodevelopment in Early Childhood: A National Retrospective Cohort Study in China

**DOI:** 10.3389/fped.2022.860192

**Published:** 2022-05-31

**Authors:** Jing Hua, Anna L. Barnett, Yao Lin, Hongyan Guan, Yuanjie Sun, Gareth J. Williams, Yuxuan Fu, Yingchun Zhou, Wenchong Du

**Affiliations:** ^1^Shanghai First Maternity and Infant Hospital, Tongji University School of Medicine, Shanghai, China; ^2^Centre for Psychological Research, Oxford Brookes University, Oxford, United Kingdom; ^3^Haikou Hospital of the Maternal and Child Health, Hainai, China; ^4^Capital Institute of Pediatrics, Beijing, China; ^5^School of Social Sciences, Nottingham Trent University, Nottingham, United Kingdom; ^6^KLATASDS-MOE, School of Statistics, East China Normal University, Shanghai, China; ^7^NTU Psychology, Nottingham Trent University, Nottingham, United Kingdom

**Keywords:** gestational age, early and post-term delivery, neurodevelopment, early childhood, national representative sample

## Abstract

**Background:**

The association between preterm birth and neurodevelopmental delays have been well examined, however, reliable estimates for the full range of gestational age (GA) are limited, and few studies explored the impact of post-term birth on child development.

**Objective:**

This study aimed to examine the long-term neuropsychological outcomes of children born in a full range of GA with a national representative sample in China.

**Methods:**

In this retrospective population-based cohort study, a total of 137,530 preschoolers aged 3–5 years old (65,295/47.5% females and 72,235/52.5% males) were included in the final analysis. The Ages and Stages Questionnaires-Third Edition (ASQ-3) was completed by parents to evaluate children's neurodevelopment. The associations between GA and neurodevelopment were analyzed by a generalized additive mixed model with thin plate regression splines. Logistic regression was also conducted to examine the differences in children's development with different GAs.

**Results:**

There was a non-linear relationship between GA and children's neurodevelopmental outcomes with the highest scores at 40 weeks gestational age. The adjusted risks of GAs (very and moderately preterm, late-preterm, early-term, and post-term groups) on suspected developmental delays were observed in communication (OR were 1.83, 1.28, 1.13, and 1.21 respectively, each *p* < 0.05), gross motor skill (OR were 1.67, 1.38, 1.10, and 1.05 respectively, each *p* < 0.05), and personal social behavior (OR were 1.01, 1.36, 1.12, and 1.18 respectively, each *p* < 0.05). The adjusted OR of very and moderately preterm, late-preterm, and early-term were observed in fine motor skills (OR were 1.53, 1.22, and 1.09 respectively, each *p* < 0.05) and problem-solving (OR were 1.33, 1.12, and 1.06 respectively, each *p* < 0.05).

**Conclusion:**

GAs is a risk factor for neurodevelopmental delays in preschoolers after controlling for a wide range of covariates, and 40–41 weeks may be the ideal delivery GA for optimal neurodevelopmental outcomes. Close observation and monitoring should be considered for early- and post-term born children as well as pre-term children.

## Background

Neurodevelopmental or neurobehavioral disorders are defined as behavioral and cognitive disorders that become apparent in early childhood and involve significant difficulties in the acquisition and execution of specific intellectual, motor, cognition, or social functions (1). Various communication disorders affecting speech and language, Autism Spectrum Disorder (ASD), Attention Deficit Hyperactivity Disorder (ADHD), Learning Disorders (LD), and motor disorders such as Developmental Coordination Disorder (DCD) are major neurodevelopmental disorders.

The prevalence, as a group, for these neurodevelopmental disorders range from 5 to 20% in the general population (2, 3), however, the etiology of neurodevelopmental disorders is complex and cannot be elucidated in a large percentage of cases (4). Neurodevelopmental disorders usually have their onset from infancy and can persist over the lifespan (5). Unfortunately, clinical symptoms of these developmental delays can be difficult to identify in early childhood, and the early diagnosis of neurodevelopmental disorders can be difficult to make, which leads to a postponement of intervention and treatment (6).

The long-term neurodevelopmental problems associated with preterm birth have been well examined. It has been indicated that very and moderately preterm birth (<34 weeks gestation) is associated with neurodevelopmental delays (7–9). Late-preterm infants (34–36 weeks) have also been reported to be more likely to have lower intellectual and psychomotor developmental assessment scores than those born full-term (10–12).

However, few studies have focused on the effects of gestational on neurodevelopmental delays age within the full-term range (37–41 weeks). For example, studies have shown that early-term infants (37–38 weeks) are at a higher risk of neurodevelopmental delays (6, 13–15). and have more complications and higher mortality than full-term children (6, 16). Previous data has also indicated that longer gestation is associated with better cognitive and psychomotor development after controlling for the effect of potential confounds such as social-economic status (SES) and home environment (17–19). Evidence also suggests that cognitive abilities can improve with a longer gestational age toward term, reaching a peak at 40 weeks before decreasing toward late-term (40–41 weeks) (20–22). However, many studies are limited by missing data (23, 24). Inconsistent results were also reported and some studies did not find significant effects in several neurodevelopmental domains between late-term and full-term children (25, 26). Most studies remain underpowered to assess whether there is continued decline in these neurodevelopmental domains with late GA.

More importantly, very little is known about the neurodevelopmental outcomes of post-term born children (>41 weeks). Although it is often assumed that full-term and post-term births are homogeneous in terms of health outcomes, post-term delivery has been reported to be associated with an increased risk of intrapartum and postpartum obstetric complications and has a high rate of perinatal morbidity and mortality (27, 28). Post-term birth is associated with significant cognitive delays compared with full-term counterparts (29). Children born post-term were more likely to have emotional and behavioral problems assessed at both 18 and 36 months old compared to their full-term counterparts (8, 30). Post-term children also presented increased risk and symptomatology of ASD (8, 31) and ADHD (32) during childhood. Evidence also suggested that post-term children were more likely to have school difficulties (33) and worse school performance (22).

Therefore, in this population-based retrospective study, using a large nationally representative sample, we systematically examined the association between a full range of GAs and the neurodevelopment of children aged 3–5 years in five neurodevelopmental domains (communication, gross motor skills, fine motor skills, problem-solving, and personal-social behavior). We hypothesized that a higher risk of neurodevelopmental delays may occur in preterm (<37 weeks), early-term (37–38 weeks), and post-term (>41weeks) children. We aimed to 1) explore a linear or non-linear relationship between GA at birth and neurodevelopment; 2) analyze the strength of effects in preterm, early-term, and post-term born children when compared with full-term born children.

## Methods

### Participants

In this national retrospective cohort study, a stratified cluster sampling plan was used to ensure that the participants included in the current study were representative of the Chinese population. Data from the 2018–2019 National Census in China provided the basis for the stratification by geographic region, age, sex, and social-economic status (SES). Government-supported maternity and children's health centers in each city were selected to invite their local kindergartens to participate in the study. Class teachers were responsible for distributing the notification to parents to complete the online questionnaire; researcher contact information was provided in case parents had queries. An electronic questionnaire system was used to enhance the quality of the data by allowing the inclusion of pop-up instructions, error messages, links to further information, and to set conditions to ensure participants could not skip questions. A data coordination center was established to take charge of establishing, managing, and maintaining the database and website, and coordinating among health centers.

**Figure 1:**
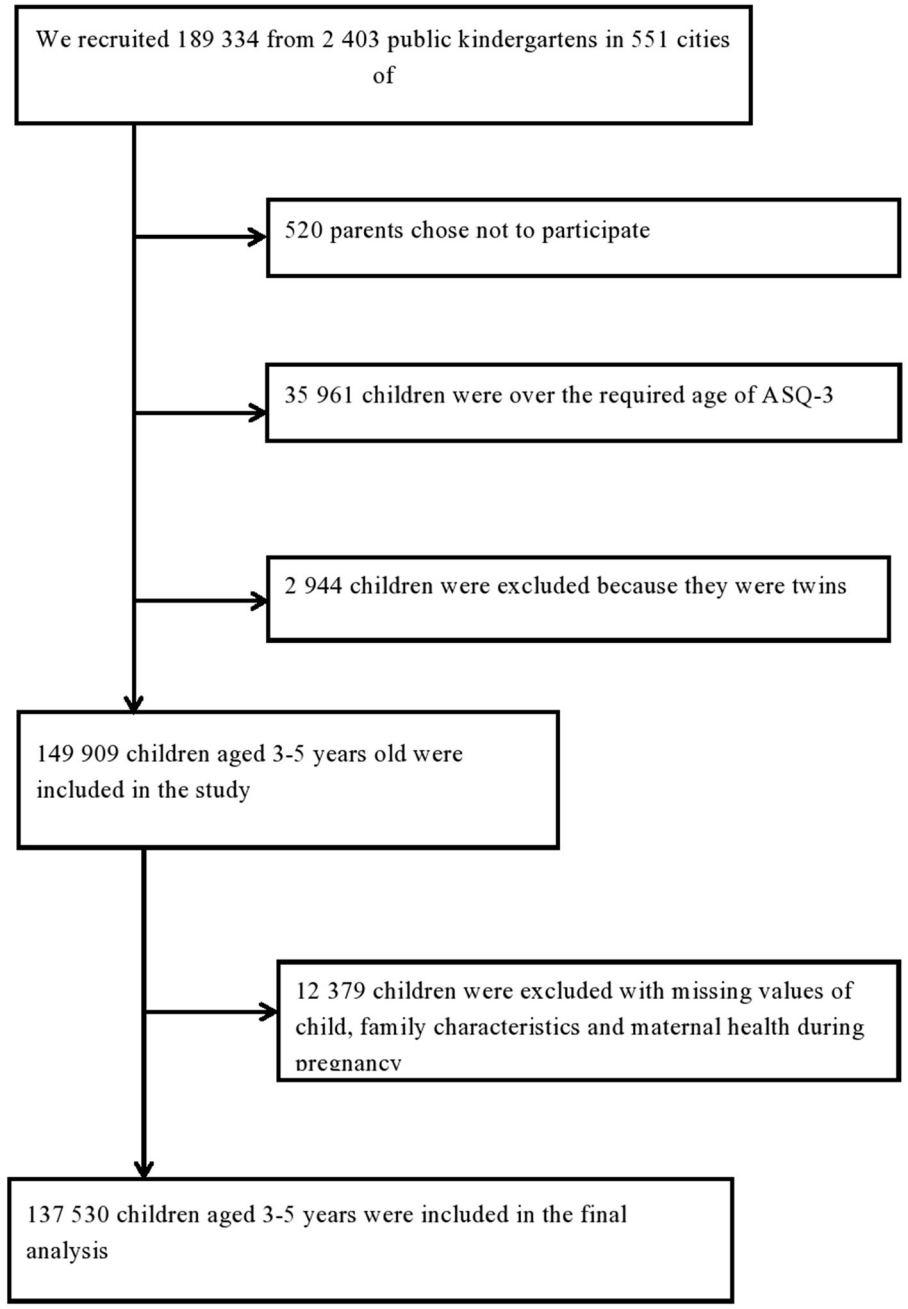
Flowchart of the study population.

Only mainstream schools and nurseries were included in the study. Children with severe visual, hearing, intellectual impairments (according to examinations that took place before starting kindergarten), or other severe developmental disorders who were required to attend special education schools/nurseries – according to local regulations – were not included in the current study. A total of 2,403 kindergartens in 551 cities of China were invited to the study from April 2018 to December 2019. Of these participants, 149 909 children with singleton delivery aged 3–5 years old were included in the study. However, children with missing covariates (child, family, and maternal characteristics) were excluded. Finally, 137,530 children with completed questionnaires were included in the final analysis (Figure 1).

The study was approved by the Ethics Committee of Shanghai First Maternity and Infant Hospital (KS18156). The parents had given online consent to participate in the study before completing the online questionnaire. All information acquired was kept confidential and was only accessible by the researchers.

### Outcomes

The preschooler's neurodevelopmental outcomes were assessed using the Ages & Stages Questionnaires-Third Edition (ASQ-3), completed by a parent of the child. This has been widely recognized and praised as a screening tool and has been validated in more than 70 languages and dialects including Chinese (34, 35). The Chinese version of ASQ-3 has fair reliability and validity in the Chinese population (36) with the Cronbach's alpha coefficient for the whole measure of 0.8.

As a screening tool designed to assess developmental status between 1 and 66 months of age, the ASQ-3 consists of 21 age-versions, each consisting of six questions per developmental domain (problem-solving, fine motor skills, gross motor skills, communication, and personal-social behavior) concerning key age-specific developmental milestones (37, 38). The scores of each domain were summed by the scores of their corresponding items. The higher score of each domain indicates the better performance of the child. According to the cutoff provided by the ASQ-3 user's guide, (39) a score that is more than two standard deviations below the mean indicates Suspected Developmental Delay (SDD) in each domain.

### Predictors

Gestational age at birth was obtained from the mother's medical records, which was based on ultrasound examination and date of last menstrual period (LMP). Gestational weeks were divided into six categories according to previous literature (32). Full-term birth was defined as 39–40 weeks gestation. The other five categories were: very preterm and moderately preterm (<34 weeks), late-preterm (34–36 weeks), early-term (37–38 weeks), late-term (41 weeks) and post-term (>41 weeks).

### Other Variates

We included the child, family, and maternal health characteristics as variables to control for when exploring the association between GA and children's ASQ-3 scores, based on the literature review (Supplementary Figure 1). All variables as shown in the columns in Table 1 were controlled for in the current study. Most of these variables were dichotomized into “yes” or “no”; BMI is an indicator of obesity which is based on height and weight [BMI = weight(kg)/height(m)] (40). Other developmental disorders included autism spectrum disorder, attention-deficit/hyperactivity disorder, learning disorders, etc. Family structures were classified into three types: three-generation (or more) family, nuclear family, and single-parent family. The “three-generation (or more) family” refers to the child living with his/her parents and grandparents, which is a traditional family structure in China; “nuclear family” refers to the child living only with his/her parents, and “single mother or father” means the child lives with one of his/her parents. We divided maternal age into three age bands: “ <30”, “30–34” and “>34” years old according to the literature (41). Higher education of mother or father refers to tertiary education leading to the award of an academic degree. Maternal complications of pregnancy and delivery were defined according to the International Classification of Diseases-Revision 10 (ICD-10). The classification is defined as having one of the following maternal complications during pregnancy, these include: vaginal bleeding during pregnancy, risk of miscarriage, use of antibiotics, use of fertility drugs, intrauterine distress, fetal asphyxia.

**Table 1:** The child and family characteristics of the study population (*n* = 137,530).

**Characteristics**	**Total**	**Gender**	
		**Male** **(*n* = 72,235)**	**Female** **(*n* = 65,295)**
**Child characteristics**			
Children's age (M, SD)	4.348 (0.68)	4.356 (0.67)	4.338 (0.68)
BMI (M, SD)	15.625 (1.63)	15.783 (1.65)	15.451 (1.60)
Right handedness (*n* %)			
No	9,884 (7.19)	5,763 (7.98)	4,121 (6.31)
Yes	127,646 (92.81)	66,472 (92.02)	61,174 (93.69)
Eyesight (*n* %)			
Normal	124,053 (90.20)	65,114 (90.14)	58,939 (90.27)
Abnormal	13,477 (9.80)	7,121 (9.86)	6,356 (9.73)
Birth weight (*n* %)			
<2500g	587 (4.70)	297 (4.11)	290 (4.44)
≥2500g	136,643 (95.30)	71,938 (95.89)	65,005 (95.45)
Gestational weeks at birth (*n* %)			
<34 (very and moderately preterm)	5,366 (3.90)	2,905 (4.02)	2,461 (3.77)
34–36 (late-preterm)	12,387 (9.01)	6,809 (9.43)	5,578 (8.54)
37–38 (early-term)	35,160 (25.57)	19,364 (26.81)	15,796 (24.19)
39–40 (full-term)	69,529 (50.56)	35,733 (49.47)	33,796 (51.76)
41 (late-term)	8,362 (6.0.8)	4,045 (5.60)	4,317 (6.61)
>41 (post-term)	6,726 (4.89)	3,379 (4.68)	3,347 (5.13)
Delivery mode			
Vaginal delivery	72,169 (52.48)	37,018 (51.25)	35,151 (53.83)
Delivery with cesarean section	65,361 (47.52)	35,217 (48.75)	30,144 (46.17)
NICU admission			
No	123,852 (90.05)	64,548 (89.36)	59,304 (90.82)
Yes	13,678 (9.95)	7,687 (10.64)	5,991 (9.18)
Other developmental disorders^a^			
No	136,482 (99.24)	71,606 (99.13)	65,876 (99.36)
Yes	1,048 (0.76)	629 (0.87)	419 (0.64)
**Family characteristics**			
Higher education of mother^b^ (*n* %)			
No	63,093 (45.88)	33,568 (46.47)	29,525 (45.22)
Yes	74,437 (54.12)	38,667 (53.53)	35,770 (54.78)
Higher education of father ^b^ (*n* %)			
No	64,404 (46.83)	34,177 (47.13)	30,227 (46.29)
Yes	73,126 (53.17)	38,058 (52.69)	35,068 (53.71)
Mother's occupation (*n* %)			
Employed	85,254 (61.99)	45,082 (62.41)	40,172 (55.62)
Unemployed	52,276 (38.01)	27,153 (37.59)	25,123 (34.78)
Father's occupation (*n* %)			
Employed	108,383 (78.81)	56,998 (78.91)	51,385 (78.70)
Unemployed	29,147 (21.19)	15,237 (21.09)	13,910 (21.30)
Family annual per-capita income (RMB)^c^ (*n* %)			
Below	26,808 (19.49)	13,910 (19.26)	12,898 (19.75)
Above or equal to	110,722 (80.51)	58,325 (80.74)	52,397 (80.25)
Family structure (*n* %)			
Single families	3,404 (2.38)	1,718 (2.38)	1,686 (2.58)
Nuclear families	85,510 (62.17)	45,090 (62.42)	40,420 (61.91)
Extended families	48,616 (35.35)	25,427 (35.20)	23,189 (35.51)
The number of children in the family (*n* %)			
One	61,431 (44.67)	31,351 (43.40)	30,080 (46.07)
Two or more	76,099 (55.33)	40,884 (56.60)	35,215 (53.93)
**Maternal health during pregnancy**			
Maternal age at delivery (*n* %)			
<30	41,877 (30.45)	21,829 (30.22)	20,048 (30.70)
30–34	66,432 (48.30)	35,049 (48.52)	31,383 (48.07)
≥35	29,221 (21.25)	15,357 (21.26)	13,864 (21.23)
Smoking or passive smoking during pregnancy (*n* %)			
No	99,274 (72.18)	52,101 (72.13)	47,173 (72.25)
Yes	38,356 (27.82)	20,134 (27.87)	18,122 (27.75)
Maternal complications during pregnancy^d^ (*n* %)			
No	131,064 (95.30)	68,910 (95.40)	62,154 (95.19)
Yes	6,466 (04.70)	3,325 (4.60)	3,141 (4.81)

a *Other developmental disorders included autism spectrum disorder, attention-deficit/hyperactivity disorder, learning disorders*.

b *Higher education of mother or father refers to tertiary education leading to the award of an academic degree*.

c *The national average family per-capita income of the year before the survey time*.

d *Having one of the following maternal complications during pregnancy including gestational diabetes, hypertensive disorders, vaginal bleeding during pregnancy, at risk of miscarriage, use of antibiotics, use of fertility drugs, intrauterine distress, fetal asphyxia*.

### Statistical Analysis

We used a generalized additive mixed model with thin plate regression splines to explore the non-linear relationship between gestational weeks and ASQ-3 scores. Based on the a stratified cluster sampling plan, a multi-level model was used which allows for grouping of child outcomes within nurseries and included residuals at the child and nursery school level, so as to represent the unobserved school characteristics that affect child outcomes. We hypothesized that there was no interaction between kindergartens and ASQ-3 scores, so we used the random intercept in the multilevel model. A mixed model utilizing a random intercept was used to investigate the associations of the different GA categories with all five sub-scores from the ASQ-3 when compared with full-term birth. A multilevel logistic regression model was also used to determine the strength of association for different GA categories and suspected neurodevelopmental delay in each domain of the ASQ-3 (0 = typically developing; 1 = SDD). A *p*-value less than 0.05 was denoted as statistically significant. LMER and GLMER procedure with R 4.0.1 was used for data statistical analysis.

## Results

Of the 137,530 children aged 3–5 years old, 65,295 (47.48%) were females, and 72,235 (52.52%) were males. A total of 69,529 (50.56%) were full-term births (39–40 weeks); 5,366 (3.90%) were very and moderately preterm (<34 weeks), 12,387 (9.01%) were late preterm (34–36 weeks), 35,160 (25.57%) were early-term (37–38 weeks), 8 362 (6.08%) were late-term (41 weeks), and 6,726 (4.89%) were post-term (>41 weeks).

The scores of all five domains of the ASQ-3 were sorted by GA category (Table 2). Preterm and early-term children showed significantly lower scores in all five domains of the ASQ-3 compared with full-term children. For late-term children, their scores in gross motor skills and communication were significantly higher than those of full-term children, however, their scores in problem-solving, fine motor skills and personal-social behavior were better than early-term but worse than full-term children. All five ASQ-3 sub-scores for post-term children were significantly lower than full-term children but higher than preterm children.

**Table 2:** ASQ-3 scores for each domain sorted by gestational age at birth (*n* = 137,530).

	**ASQ-3**				
**Gestational age**	**Communication**	**Gross motor**	**Fine motor**	**Problem-solving**	**Personal-social behavior**
ASQ-3 scores Mean (SD)					
<34 (very and moderately preterm)	51.69 (11.96)	47.27 (13.76)	46.58 (14.11)	51.85 (11.89)	52.98 (10.93)
34–36 (late-preterm)	52.74 (10.59)	48.08 (13.07)	47.27 (13.59)	52.95 (10.68)	54.01 (9.59)
37–38 (early-term)	53.82 (9.96)	49.82 (12.11)	48.45 (12.94)	53.95 (9.84)	54.49 (8.92)
39–40 (full-term)	54.34 (9.56)	50.47 (11.74)	49.14 (12.66)	54.34 (9.53)	54.82 (8.67)
41 (late-term)	54.45 (9.27)	50.68 (11.54)	49.12 (12.57)	54.34 (9.35)	54.78 (8.64)
>41 (post-term)	53.11 (10.92)	49.33 (12.67)	48.49 (13.04)	53.40 (10.56)	54.43 (9.44)
Neurodevelopmental delay *n* (%)					
<34 (very and moderately preterm)	433 (8.09)	987 (18.39)	322 (6.00)	261 (4.86)	418 (7.79)
34–36 (late-preterm)	821 (6.63)	2,160 (17.44)	641 (5.17)	481 (3.88)	773 (6.24)
37–38 (early-term)	1,886 (5.36)	4,546 (12.93)	1,447 (4.12)	1,094 (3.11)	1,680 (4.78)
39–40 (full-term)	3,189 (4.58)	8,066 (11.60)	2,532 (3.64)	2,081 (2.99)	2,938 (4.23)
41 (late-term)	346 (4.14)	925 (11.06)	302 (3.61)	230 (2.75)	356 (4.26)
>41 (post-term)	464 (6.90)	982 (14.60)	291 (4.30)	255 (3.80)	368 (5.47)

**Figure 2:**
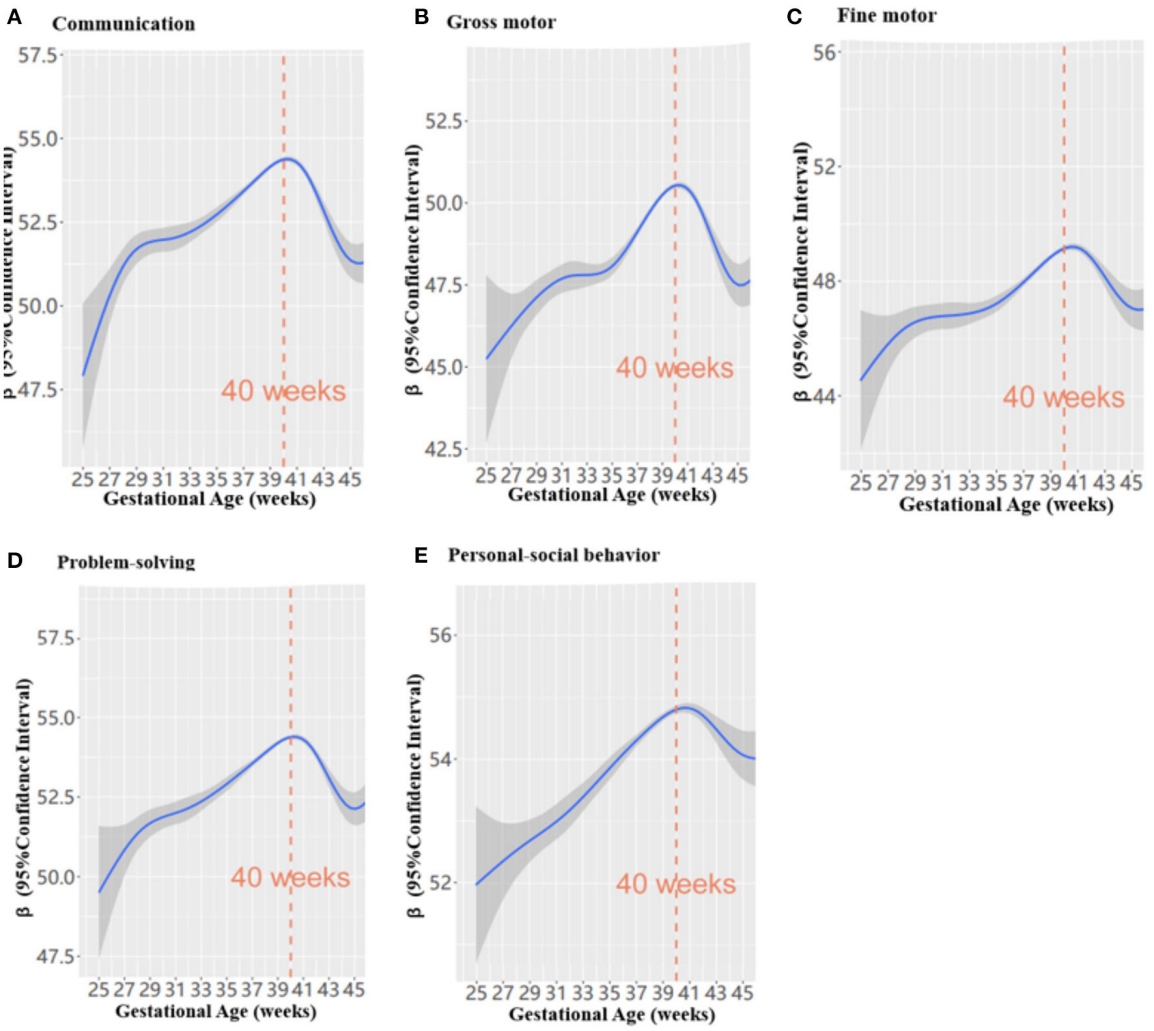
The non-liner relationship between gestational weeks and ASQ-3 scores in preschoolers using a generalized additive mixed model (*n* = 137 530); **(A–E)** refers to different domains.

We observed a non-linear relationship of GA with scores of problem-solving, gross motor skill, fine motor skill, communication and personal-social behavior in the ASQ-3 (Figure 2). We found that the ASQ-3 scores in all five domains increased with rising gestational weeks before 40 weeks, and decreased with the fall of gestational weeks after 40 weeks (each *p* < 0.05). Children who were born at 40 weeks gestation performed the best in problem-solving, gross motor skills, fine motor skills, communication and personal-social behavior.

The crude and adjusted association between gestational age and scores on the ASQ-3 in preschoolers are shown in Table 3. Compared with the full-term group, associations between very and moderately preterm, late-preterm, early-term, late-preterm, and post-term groups in problem-solving (β were −0.19, 0.87, 0.25, 0.138, −0.56, each *p* < 0.05), gross motor skill (β were −2.02, −1.18, −0.39, −0.31, each *p* < 0.05), fine motor skill (β were −0.16, −0.93, −0.46, −0.10, each *p* < 0.05), communication (β were −1.84, −0.81, −0.31, −0.64 respectively, each *p* < 0.05), and personal-social behavior (β were −1.57, −0.59, −0.28, 0.07, −0.23, each *p* < 0.05) were found when adjusting for the child, family and maternal health characteristics. However, there were no statistically significant differences between late-term birth and ASQ-3 sub-scores when compared with full-term birth (each *p* > 0.05).

**Table 3:** The association between gestational age and ASQ-3 scores in preschoolers (*n* = 137,530).

**Gestational week**	**Communication**		**Gross motor**		**Fine motor**		**Problem–solving**		**Personal social behavior**	
	**Crude β**	**Adjusted β^a^**	**Crude β**	**Adjusted β^a^**	**Crude β**	**Adjusted β^a^**	**Crude β**	**Adjusted β^a^**	**Crude β**	**Adjusted β^a^**
	**(95% CI)**	**(95% CI)**	**(95% CI)**	**(95% CI)**	**(95% CI)**	**(95% CI)**	**(95% CI)**	**(95% CI)**	**(95% CI)**	**(95% CI)**
<34 (very and moderately preterm)	−2.64 *** (−2.92, −2.37)	−1.84*** (−2.12, −1.56)	−3.20 *** (−3.53, −2.86)	−2.02 *** (−2.36, −1.69)	−2.56*** (−2.92, −2.20)	−0.16*** (−1.95, −1.24)	−2.50 *** (−2.77, −2.22)	−0.19*** (−1.06, −0.68)	−1.84*** (−2.09, −1.59)	−1.57*** (−1.82, −1.32)
34–36 (late–preterm)	−1.59 *** (−1.78, −1.41)	−0.80*** (−1.00, −0.61)	−2.38*** (−2.62, −2.15)	−1.18*** (−1.41, −0.95)	−1.86*** (−2.11, −1.62)	−0.93*** (−1.18, −0.69)	−0.14*** (−1.58, −1.20)	−0.87*** (−1.06, −0.68)	−0.81*** (−0.98, −0.63)	−0.59*** (−0.76, −0.41)
37–38 (early–term)	−0.51*** (−0.64, −0.39)	−0.31*** (−0.44, −0.18)	−0.65 *** (−0.81, −0.50)	−0.39*** (−0.54, −0.24)	−0.68*** (−0.85, 0.52)	−0.46*** (0.62, −0.29)	−0.39*** (−0.52, −0.27)	−0.25*** (−0.38, −0.12)	−0.33*** (−0.45, −0.22)	−0.28*** (−0.40, −0.17)
39–40 (full–term)	Reference	Reference	Reference	Reference	Reference	Reference	Reference	Reference	Reference	Reference
41 (late–term)	0.11 (−0.11, 0.34)	0.12 (−0.11, 0.34)	0.21 (−0.06, 0.48)	0.11 (−0.16, 0.38)	−0.01 (−0.31, 0.28)	0.11 (−0.18, 0.40)	0.001 (−0.22, 0.22)	0.13 (−0.09, 0.35)	−0.04 (−0.24, 0.17)	0.07 (−0.14, 0.027)
>41 (post–term)	−1.22*** (−1.47, −0.98)	−0.64 *** (−0.89, −0.39)	−1.14*** (−1.44, −0.83)	−0.31* (−0.61, −0.01)	−0.65 *** (−0.97, −0.32)	−0.10 (−0.42, 0.22)	−0.94*** (−1.19, −0.69)	−0.56*** (−0.81, −0.32)	−0.0.39*** (0.61, −0.17)	−0.23* (−0.46, −0.01)

a *p < 0.05, **p < 0.01, ***p < 0.001.

*^b^Adjusting for the child, family characteristics and maternal health during pregnancy*.

**Figure 3:**
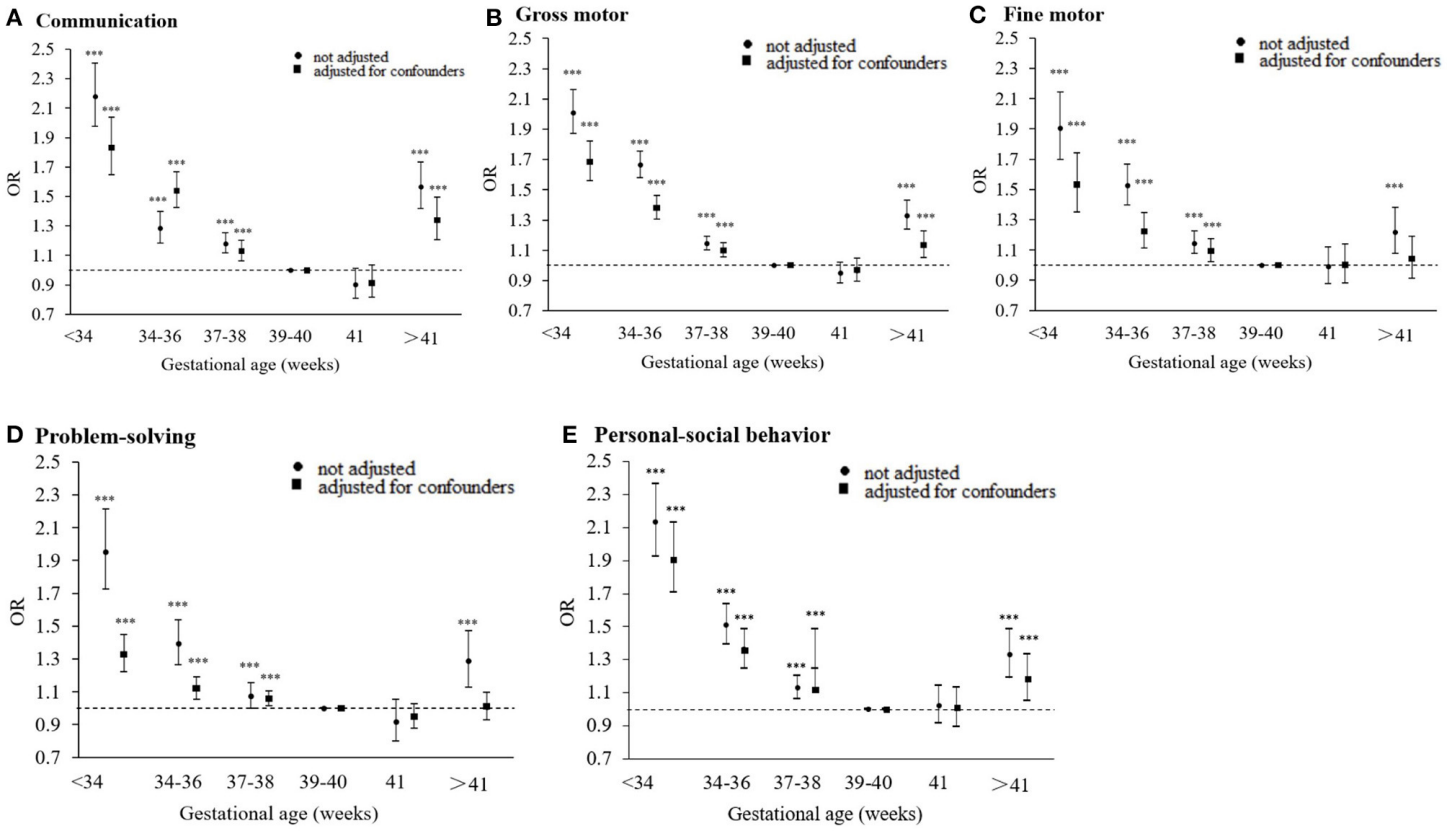
Association between gestational age (weeks) and neurodevelopmental delays in preschoolers when adjusting or not adjusting for child, family characteristics and maternal health during pregnancy (*n* = 137 530); **(A–E)** refers to the different domains (***p < 0.001).

Associations were also found between GA categories and the risk of SDD when adjusting or not adjusting for the child, family, and maternal health characteristics (Figure 3). The adjusted risks of GA categories (very and moderately preterm, late-preterm, early-term, and post-term groups) on preschoolers' suspected neurodevelopmental delays were observed in communication (OR were 1.83, 1.28, 1.13, and 1.21 respectively, each *p* < 0.05), gross motor skill (OR were 1.67, 1.38, 1.10, and 1.05 respectively, each *p* < 0.05), and personal-social behavior (OR were 1.01, 1.36, 1.12, and 1.18 respectively, each *p* < 0.05). When adjusting for the child, family, and maternal health characteristics, we found the associations of GA categories (very and moderately preterm, late-preterm, and early-term) with fine motor skills (OR were 1.53, 1.22, and 1.09 respectively, each *p* < 0.05) and problem-solving (OR were 1.33, 1.12, and 1.06 respectively, each *p* < 0.05). However, no statistically significant difference was found for late-term birth on SDD when compared with full-term birth (each *p* > 0.05).

## Comment

### Principal Findings

To our knowledge, this is the first study to systematically examine the associations between GA across the full range of gestation and neurodevelopment based on a national population-based sample in China. A non-linear regression model was found to best explain the association between GA and children's neurodevelopment at 3–5 years old. Children's neurodevelopmental outcomes benefit from longer gestation before 40 gestational weeks but decrease after 40 gestational weeks. The novel findings of our study were that a higher risk of neurodevelopmental delays was found in early-term and post-term birth when compared with full-term birth.

### Strengths and Limitations

The strengths of our study are that we involved a large national representative sample to examine the effects of GA across a full range and neurodevelopmental outcomes with a focus on early-term and post-term birth. We also included a comprehensive number of possible confounders including the child, family, and maternal health characteristics. Some limitations should also be considered in the current study. First, it should be noted that children with severe visual, hearing, intellectual impairments, or other severe developmental disorders who were required to attend special education schools/nurseries were not included in the current study. The previous studies (42, 43) suggested a strong association between preterm birth and special education needs in children with severe impairments, further studies should also include children with special education needs and examine the associations for children with severe impairments across the full range of gestation. Second, possible sample selection bias and recall bias might also exist: the current study only included children attending nurseries but did not include children who did not attend nurseries, and the accuracy of information provided using the ASQ-3 is dependent on the subjective judgment of parents. In addition, based on a retrospective cohort, our research may provide limited information when it comes to uncovering causal associations. In future studies, in-depth evaluation of developmental delays could be made to further verify the results of this study. Further research is needed to explain the mechanisms linking early- and post-term birth to neurodevelopmental delays. Randomized controlled trials could also be considered to provide evidence of the causal association between gestational age and neuropsychological development (44).

### Interpretation

We observed an inverted U-shaped relationship between GA and neurodevelopmental outcome as measured by the ASQ-3 (in all five domains: problem-solving, fine motor skills, gross motor skills, communication, and personal-social behavior). We found that neurodevelopmental outcomes improved with the rising of gestational weeks before 40 gestational weeks, and decreased after 40 gestational weeks. The findings were consistent with previous studies that 40 gestational weeks at birth is the optimal GA for children's health (13, 23, 45–47). Similar evidence reported that gestation from 37 to 41 gestational weeks benefits both the cognitive and motor development of infants (19). Evidence from neuroimaging studies has indicated that a longer gestational duration was associated with region-specific increases in gray matter density (48) and a more efficient neural network (49). In particular, late gestational cerebellar growth is rapid as a linear increase in total gray matter volume of 1.4% per week can be seen from 29 to 41 gestational weeks (18, 50), and approximately 50% of the increase in cortical volume occur between 34 and 40 gestational weeks (51). Imaging studies have also suggested that a longer gestational duration was associated with region-specific increases (temporal lobes) in gray matter density (48), and children born at term with a shortened GA were observed to have a reduced neural network efficiency in the posterior medial cortex, including the precuneus, cuneus, and superior parietal regions (49). Another study has also suggested the benefits of longer gestation in temporal cortex development, one of the latest brain regions to mature during gestation, which is related to cognitive development (52).

For premature birth, we observed the strongest associations of very and moderately preterm with SDD, which is consistent with the reports of previous studies (8, 32, 53). Moreover, our results provide reliable evidence that late-preterm children also have an increased risk of neurodevelopmental delays based on a large national sample. The association between preterm birth and brain development has been well examined, and previous studies have shown that preterm birth is associated with altered hemispheric connections (54), loss of gray/white matter (55, 56), disrupted myelination, abnormal cortical folding (57, 58) and thalamocortical abnormalities (59). In particular, the abnormal connection and development of white matter and specific functional areas (especially the frontal cortex, corpus callosum, thalamus, and cingulate gyrus) are important causes of neurodevelopmental delay in preterm children (60). In addition, shortened gestation indicates that the fetus may be exposed to unfavorable intrauterine conditions. This may lead to modifications in the nervous system and an adjusted developmental trajectory to ensure survival for the fetus (61).

With our analysis, we found that even within the full-term range of GA (37–41 weeks), there were still significant differences among children born with different GAs. The early-term children (37–38 weeks) had a decreased neurodevelopmental outcomes and a higher risk of SDD when compared with their full-term counterparts. The results are consistent with previous studies in which early-term children have affected performance in cognition (14), motor (62), and language development (63, 64) during the early years of life. Our study has confirmed that such negative effects can last until 3–5 years of age, and also exists in social behaviors which are different from what has previously been reported in the literature (65). Our results support the findings from previous studies which emphasized the importance of the last few weeks of gestation (37 th and 38 th week) (66) The last few weeks are an important period for the development of the neurological system and the total volume of gray and white matter increases significantly between 35 and 41 weeks of gestation (67). Even at 38 weeks of gestation, the brain is still only 90% of the full-term weight (68).

More importantly, we found a stronger association between post-term birth and SDD when compared with late-preterm and early-term birth. In recent years, several studies have highlighted the significant negative effect on cognitive delays (29), emotional and behavioral problems (8, 30), and developmental disorders of post-term birth (8, 31). Our study confirms the negative effect of post-term birth on neurodevelopment in all five domains including problem-solving, communication, fine motor skills, gross motor skills, and personal-social behavior. The mechanisms underlying the negative effect of post-term birth on children's neurodevelopment can be explained by obstetric or neonatal complications marked by late-preterm birth, or a combination of both. First, a larger weight baby at birth, associated with a longer GA, has a higher risk for perinatal problems such as prolonged labor, which can cause a perinatal lack of oxygen, which has been shown to be associated with behavioral problems (69). Second, post-term birth is related to uteroplacental insufficiency (70), and compared with full-term birth, the placenta in post-term birth offers fewer nutrients and less oxygen which is associated with abnormal emotional and behavioral development (30). Finally, previous studies have shown that the placental secretion of corticotrophin-releasing hormone (71), the principal regulator of the maternal and fetal hypothalamic–pituitary–adrenal axis (72) is lower in women who deliver post-term than full-term. These neuroendocrine abnormalities might increase children's vulnerability to emotional and behavioral problems later in life (30).

## Conclusions

Our study found a non-linear relationship between GA and children's neurodevelopmental outcomes, with 40 gestational weeks being the most optimal GA for children's neurodevelopment. Before 40 gestational weeks, neurodevelopmental outcomes increased with gestational weeks; and after 40 gestational weeks, neurodevelopmental outcomes decreased with gestational weeks. In addition to preterm birth, the risks of suspected neurodevelopmental delays increased in children born early-term and post-term. Our results can inform clinical professionals, parents, and teachers who should not neglect the long-term neurodevelopmental risks of being born in the early-term and post-term gestational period. The results of this study can inform clinical professionals when considering the optimal timing of birth during the full-term period. Elective birth interventions should only be recommended if the risk of continuing the pregnancy is higher than the delivery (post-term birth). The same as preterm birth, early-term and post-term born children also need close follow-up and additional assessment for detecting potential neurodevelopmental delays.

## Data Availability Statement

The original contributions presented in the study are included in the article/Supplementary Material, further inquiries can be directed to the corresponding authors.

## Ethics Statement

The studies involving human participants were reviewed and approved by the Ethics Committee of Shanghai First Maternity and Infant Hospital (KS18156). Written informed consent to participate in this study was provided by the participants' legal guardian/next of kin.

## Author Contributions

WD and JH: access to all of the data in the study, took responsibility for the integrity of the data, the accuracy of the data analysis, and concept and design. JH, YS, and GW: acquisition, analysis, or interpretation of data. JH: drafting of the manuscript. JH, WD, AB, and GW: critical revision of the manuscript for important intellectual content. YF, YZ, and YS: statistical analysis. JH and YS: obtained funding. WD and YS: administrative, technical, or material support. WD: supervision. All authors contributed to the article and approved the submitted version.

## Conflict of Interest

The authors declare that the research was conducted in the absence of any commercial or financial relationships that could be construed as a potential conflict of interest.

## Publisher's Note

All claims expressed in this article are solely those of the authors and do not necessarily represent those of their affiliated organizations, or those of the publisher, the editors and the reviewers. Any product that may be evaluated in this article, or claim that may be made by its manufacturer, is not guaranteed or endorsed by the publisher.
