# Supplementary material for: Association of Gestational Age at Birth With Subsequent Neurodevelopment in Early Childhood: A National Retrospective Cohort Study in China
Source: Front Pediatr. 2022 May 31;10:860192. doi: 10.3389/fped.2022.860192 (PMC9194570; doi:10.3389/fped.2022.860192)
Supplement: Supplementary file 1 [file Table_1.DOCX]

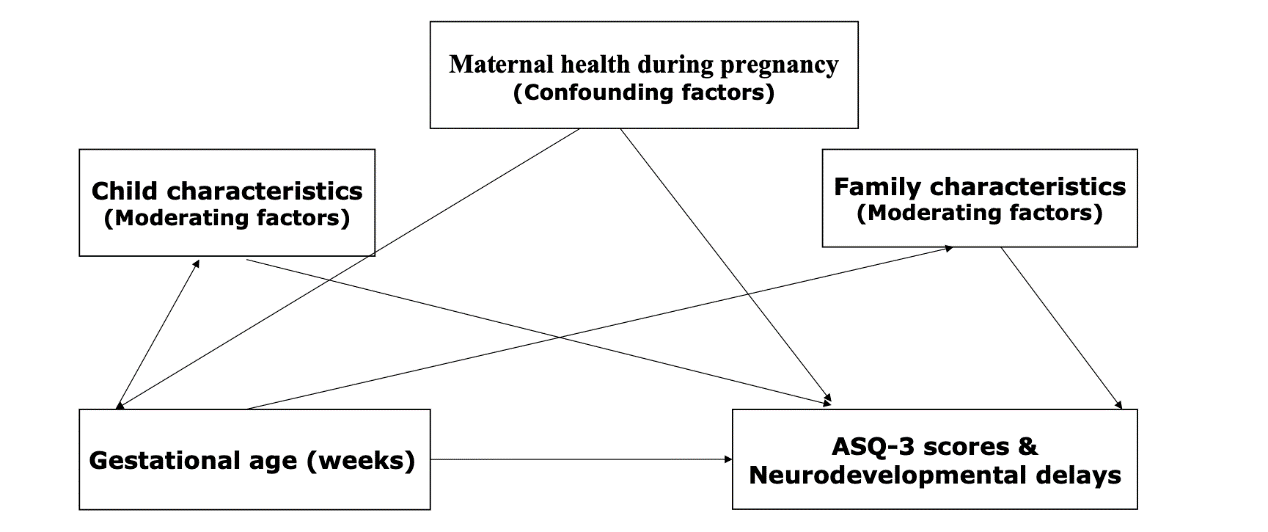


**Supplementary Figure 1.** The summarized directed acyclic graphic (DAG). The variables controlled for in the statistical analysis were summarised according to the literature. Maternal health during pregnancy variables were considered as key confounders and child and family characteristics were considered key moderating factors when investigating the effects of gestational age on neurodevelopmental outcomes and Suspected Developmental disorders.
